# Expression of phosphatase of regenerating liver (PRL)-3, is independently associated with biochemical failure, clinical failure and death in prostate cancer

**DOI:** 10.1371/journal.pone.0189000

**Published:** 2017-11-30

**Authors:** Sigve Andersen, Elin Richardsen, Mehrdad Rakaee, Helena Bertilsson, Roy Bremnes, Magne Børset, Lill-Tove Busund, Tobias Slørdahl

**Affiliations:** 1 Translational Cancer Research Group, Department Clinical Medicine, UiT, The Arctic University of Norway, Tromso, Norway; 2 Department Oncology, University Hospital of North Norway, Tromso, Norway; 3 Translational Cancer Research Group, Department of Medical Biology, UiT, The Arctic University of Norway, Tromso, Norway; 4 Department Pathology, University Hospital of North Norway, Tromso, Norway; 5 Department of Cancer Research and Molecular Medicine, NTNU - Norwegian University of Science and Technology, Trondheim, Norway; 6 Department of Urology, St. Olavs Hospital - Trondheim University Hospital, Trondheim, Norway; 7 Department of Immunology and Transfusion Medicine, St. Olavs Hospital - Trondheim University Hospital, Trondheim, Norway; 8 Department of Hematology, St. Olavs Hospital - Trondheim University Hospital, Trondheim, Norway; University of South Alabama Mitchell Cancer Institute, UNITED STATES

## Abstract

**Background:**

Prostate cancer (PC) stratification needs new prognostic tools to reduce overtreatment. Phosphatase of regenerating liver (PRL-3) is a phosphatase found at high levels in several cancer types, where its expression is associated with survival. A recent PC cell line study has shown it to be involved in PC growth and migration.

**Methods:**

We used a monoclonal antibody to evaluate the expression of PRL-3 in PC tissue of patients in an unselected cohort of 535 prostatectomy patients. We analyzed associations between PRL-3 expression and biochemical failure-free survival (BFFS), clinical failure-free survival (CFFS) and PC death-free survival (PCDFS).

**Results:**

Cytoplasmic PRL-3 staining in tumor cells was significantly correlated to expression of molecules in the VEGFR-axis, but not to the clinicopathological variables. High PRL-3 was not significantly associated with survival in the univariate analysis for BFFS (p = 0.131), but significantly associated with CFFS (p = 0.044) and PCDFS (p = 0.041). In multivariate analysis for the various end points, PRL-3 came out as an independent and significant indicator of poor survival for BFFS (HR = 1.53, CI95% 1.10–2.13, p = 0.012), CFFS (HR = 2.41, CI95% 1.17–4.98, p = 0.017) and PCDFS (HR = 3.99, CI95% 1.21–13.1, p = 0.023).

**Conclusions:**

PRL-3 is independently associated with all PC endpoints in this study. Since high PRL-3 expression also correlates with poor prognosis in other cancers and functional studies in PC support these findings, PRL-3 emerges as a potential treatment target in PC.

## Introduction

Prostate cancer (PC) is the fourth most common cancer overall and the second most common in men worldwide [1]. Presently, the identification of clinically relevant PC is challenging since overdiagnosis and overtreatment coexist, while many die of aggressive PC [2]. There are ongoing efforts to improve the identification of aggressive PC, but these efforts are hampered by the lack of useful tools. Although recent efforts, like the composite pre-biopsy STHLM3 model, are entering the field[3], the morphology grade scored by pathologists is still today the strongest predictor of aggressive disease[4]. Besides, there is currently no widely used prognostic molecular tissue markers in PC. Hence, improved prognostic and more so predictive molecular markers are urgently needed in this field.

Phosphatase of regenerating liver (PRL)-3 is a dual specificity phosphatase with ability to dephosphorylate tyrosine, serine and threonine residues. In 2001, Vogelstein’s group suggested that the PRL-3 gene (gene name: *PTP4A3*) is important for colorectal cancer metastasis as they found high levels of *PTP4A3* expression in metastases from colorectal cancer compared to non-metastatic tumors and normal colorectal epithelium[5]. Studies have found PRL-3 to be associated with epithelial-mesenchymal transition (EMT) and cancer progression[6]. Other studies have shown PRL-3 to be associated with metastatic potential and poor prognosis in a large number of cancers[7–16], as well as being upregulated in myeloma cells[17]. Due to these studies, PRL-3 has been proposed a promising biomarker for assessing tumor aggressiveness and metastatic potential[18]. In addition, targeting of PRL-3 has been proposed and several studies have recently reported endogenous suppressing proteins[19] and a new humanized antibody against PRL3 (PRL3-zumab) has been tested in orthotopic gastric tumors[20].

In PC, PRL-3 has previously been identified as a mediator of PC progression and aggressiveness in an integrated assessment of aggressiveness through gene copy number and gene expression analyses[21]. As PRL-3 is a potential cancer biomarker and biomarkers in PC are in high demand, Exploring the expression and biological role of PRL-3 in PC cells, Vandsemb et al [22] found PRL-3 mRNA to be highly expressed in PC tissue compared to benign prostate tissue, and the PRL-3 protein was expressed in both primary PCs and regional lymphatic metastasis. Further *in vitro* studies found inhibition to induce growth arrest and decreased migration of PC cancer cells. They also evaluated and found PRL-3 expression in 4/4 cases by immunohistochemistry.

To further explore PRL-3’s role in PC, we aimed to elucidate the expression profile and prognostic impact of PRL-3 in a large cohort of PC patients. Herein, we present the results using a validated PRL-3 antibody on tissue microarrays (TMAs) from a large, well described retrospective cohort with an extensive follow-up[23].

## Material and methods

### Patients, tissue micro arrays and endpoints

Patients were included after retrospective identification of 671 patients from the archives of the departments of pathology in two health regions in Norway, undergoing radical prostatectomy (RP) for adenocarcinoma of the prostate between 01.01.1995 to 31.12.2005. One-hundred and thirty-one (131) patients were excluded, due to non-available tissue blocks for re-evaluation (St. Olav n = 112, NLSH n = 3, UNN n = 15) [23]. A total of 535 eligible patients with available tissues and complete follow-up data were included in this retrospective cohort study. Two-hundred and twenty-eight (228) patients were from St. Olav Hospital/Trondheim University Hospital (St. Olav) in the Central Norway region, and 59 from Nordlandssykehuset Bodo (NLSH) and 248 from the University Hospital of North Norway (UNN), both in the Northern Norway region. In total, 435 patients were submitted to open retropubic resection and 100 patients had perineal resection.

From the cohort we constructed 12 tissue micro array (TMA) blocks. A tissue-arraying instrument (Beecher Instruments, Silver Springs, MD, USA) was used to harvest cores from formalin-fixed paraffin-embedded (FFPE) tissue blocks from included patients. Two cores were sampled from the most dedifferentiated neoplastic cell compartment, hereafter designated *tumor*. Furthermore, two cores were sampled from reactive tumor stroma, hereafter designated *stroma*. The cores were carefully inserted into paraffin blocks. Then, 4 μm sections were cut by a Micron microtome (HM355S) and affixed to glass slides prior to immunostaining and scoring.

Biochemical failure (BF) was defined as a PSA ≥ 0.4 ng/ml and BF-free survival (BFFS) was calculated as time from surgery to last follow up (FU) date or date with PSA ≥ 0.4 ng/ml. Clinical failure (CF) was defined as symptomatic, locally advanced progression or radiologically verified metastasis to bone, visceral organs or lymph nodes. Clinical failure-free survival (CFFS) was calculated as time from surgery to last fFU date without CF or to date of CF. Last follow-up update was December 2015, and calculated median follow-up of survivors was 150 months.

For more extensive information regarding patients, exclusion, definitions of variables and endpoints, see our previous report[23].

### Immunohistochemistry

TMA paraffin block sections slides were dried overnight at 37°C. PRL-3 immunohistochemical staining of the cut sections was performed using the Ventana Discovery ULTRA autostainer (Tucson, Arizona, USA). After paraffin embedded tissues were dewaxed, antigen retrieval was applied using Ventana ULTRA Cell Conditioning-1 (CC1) for 32 minutes at 95°C. Endogenous peroxidase was blocked by discovery inhibitor CM (#760–4306, Ventana) for 12 minutes. Sections were incubated with non-commercial mouse monoclonal antibody[24, 25] (kind gift from professor Qi Zeng, Agency for Science, Technology and Research (ASTAR), Singapore) with 1/50 dilution for 32 minutes at 36°C. As secondary antibody, OmniMap anti-mouse HRP (#760–4310, Ventana) was loaded for 20 minutes, followed by 8 minutes of HRP amplification. The detection chromogen was ChromoMap DAB (#760–159; Ventana). Counterstaining was performed using the hematoxylin II (#790–2208, Ventana) counterstain for 32 minutes and then with a bluing reagent for 8 minutes. Staining was performed in one single experiment and a human multiple organ (normal and malignant) tissue array was included for specificity control of antibody. Normal tonsil and liver adenocarcinoma were used as negative and positive tissue controls, respectively.

### Scoring of immunohistochemistry and cut-offs

PRL-3 expression was scored semiquantitatively. We initially explored the expression with our dedicated uropathologist (E.R.), and agreed on scoring definitions and scales. Then, two scorers (E.R, M.R) performed all scoring and reported the scores independently of each other. We sought to assess expression in applicable compartments (tumor, non-malignant epithelium and stroma) and different cell compartments (cytoplasmic, nuclear or membranous). Scorable PRL-3 expression was only possible where positivity was present in more than a minor subset. We ended up with the following scoring scale based on observed expression: A. Tumor cytoplasmic cell intensity on a four-tier scale (0 = negative, 1 = weak, 2 = intermediate, 3 = strong), and B. Tumor nuclear density on a four tier scale (0 = 0%, 1 = 0–5%, 2 = 5–50% and 3 >50% of nuclear tumor cells stained). A cut-off of 1.5 was defined for all analyses.

### Statistical analyses

We used the SPSS software version 23 (IBM SPSS Inc., Chicago, IL, USA) for statistical analyses. For the Inter-observer reliability of scoring, we used the two-way random effect model with absolute agreement. Correlations between PRL-3, previous explored markers and clinicopathological variables were assessed by the Spearman Correlation test. The log-rank test was used for testing statistical significance of difference between survival curves. Survival curves were drawn by use of the Kaplan-Meier method. The curves were terminated when less than 10% of patients were still at risk (192 months). For the multivariate analyses, we used a backward Cox regression model with a probability at 0.10 for entry and 0.05 for removal. Clinicopathological variables from the univariate analyses with a p < 0.10 were entered. The significance level defined for all analyses was p < 0.05.

### Ethics

This study was approved by the regional ethics committee, REK Nord, project application 2009/1393 (including a mandatory re-application which was formally approved 22.01.2016. The committee waived the need for patient consent for this retrospective study. The reporting of clinicopathological variables, survival data and biomarker expressions is in accordance with the REMARK guidelines.

## Results

### Expression of PRL-3

**Fig 1:**
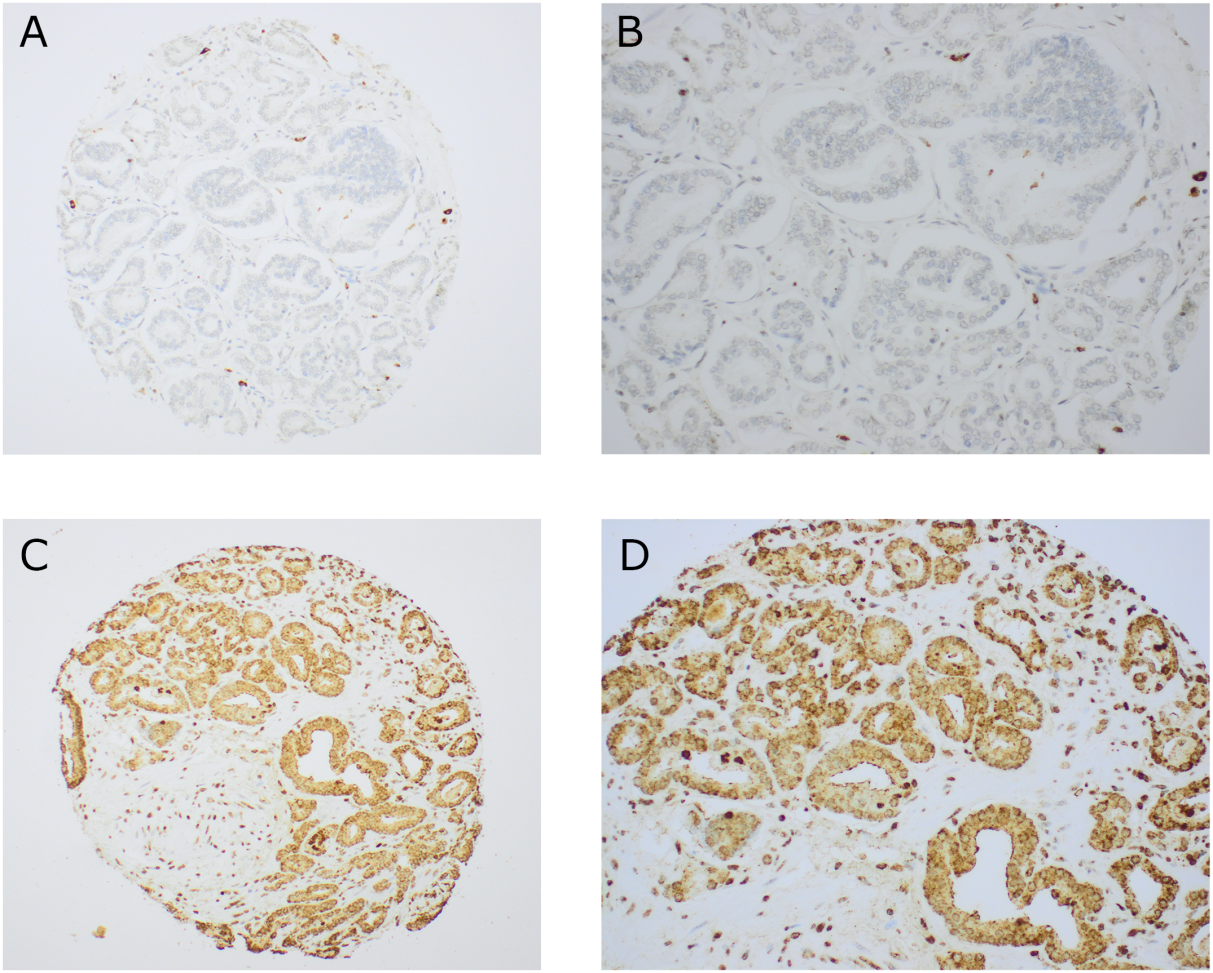
Illustrative examples of immunohistochemical staining for PRL3. A) a whole core at 200 magnification exhibiting low expression, B) An image of the same core as A at 400X magnification, C) a whole core at 200x showing high expression of PRL-3, D) an image taken 400x in the same core as C. This image also serves as an example of high expression in fibroblast nuclei.

There was specific and variable cytoplasmic staining, which when present, was frequently accompanied by a granular accentuation. There was also strong nuclear staining in a subset of tumor cells. In stroma, a small subset of fibroblasts had some nuclear staining. Most lymphocytes, when present, also had a strong nuclear staining. Expression of PRL-3 was also present in benign epithelium in this study, although its extent was not systematically evaluated. Interobserver scoring agreement was; Intraclass correlation coefficient (ICC) = 0.89 for tumor cytoplasm intensity and ICC = 0.93 for tumor cell nuclear staining. The fibroblast staining was hard to score due to very low intensity, resulting in an ICC of 0.44. Photomicrographs of low versus high expression examples of cytoplasmic tumor cell expression of PRL-3 are presented in Fig 1. For examples of IHC staining in whole tissue sections, see S1 Fig.

Of the total cohort, 397 patients had cores with morphologically verified malignant cells available for scoring. In tumor cell cytoplasm, the mean expression score was 1,25, (range 0–3) and the most prevalent score was 1 (n = 112). For tumor nuclear staining, the mean expression score was 0.48 with 0 as the most prevalent score (n = 225). For fibroblasts, only 56 had some cytoplasmic staining detected by at least one of the observers. The ICC for fibroblast scoring was weak and considered unreliable for further analyses.

### Correlations

We observed a positive and significant correlation between cytoplasmic and nuclear PRL-3 staining (*r* = 0.42, p < 0.001). There was no significant correlation with *r* > 0.1 between clinicopathological variables and cytoplasmic or nuclear PRL-3 staining. However, we found cytoplasmic PRL-3 staining to correlate to the following molecular markers previously evaluated in our cohort; tumoral VEGF-A (*r* = -0.21, p < 0.001), tumoral VEGFR-2 (*r* = 0.22, p < 0.001) and tumoral VEGFR-3 (*r* = 0.31, p < 0.001). The markers it did not correlate to were monocarboxylate trasporter 1 and 4, CD3, CD4, CD8, CD 20, CD56, CD68, CD138, PD1, progesterone receptor, estrogen receptor and aromatase. For nuclear PRL-3 staining, we found no significant correlations with *r* > 0.1.

### Univariate analyses

**Fig 2:**
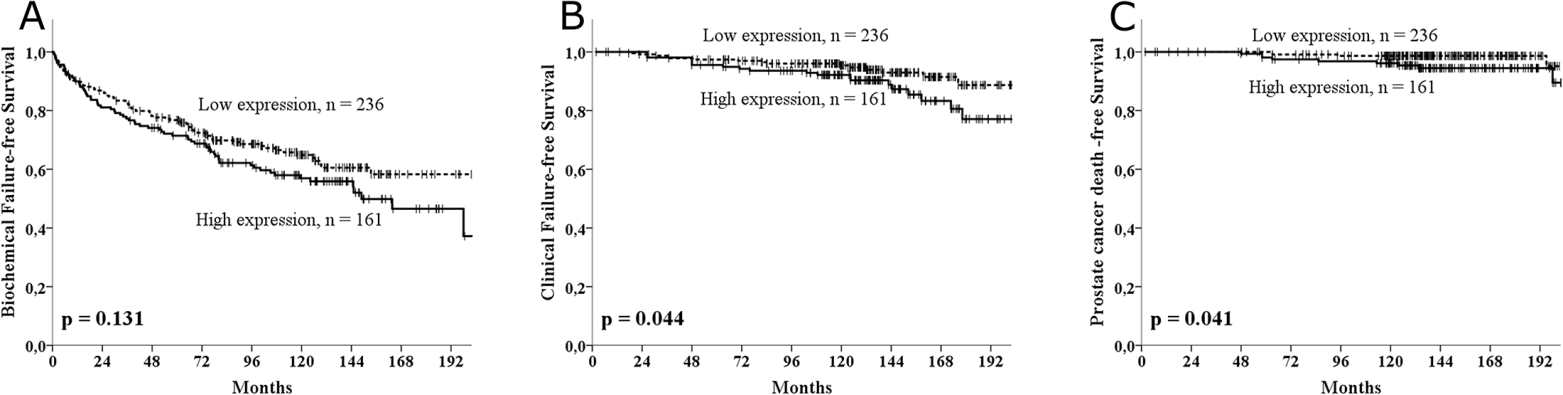
PRL-3 survival curves. Kaplan meier curves stratified by high and low expression of PRL-3 for A) biochemical failure-free survival, B) clinical failure-free survival and C) prostate cancer death free survival. The p-value is the univariate log rank p-value.

For nuclear PRL-3 expression there was no significant association to BFFS or CFFS. For cytoplasmic expression of PRL-3 we found associations between high expression of PRL-3 and poor BFFS (p = 0.131, Table 1 and Fig 2A), poor CFFS (p = 0.044, Table 1 and Fig 2B) and poor PCDFS (p = 0.041, Table 1 and Fig 2C). When exploring different cut-offs, we found a trend for worse survival for all cut-offs with variable p-values. The same tendency or significance was observed within each relevant clinicopathological subgroup (PSA, age, Gleason, pTstage, Tumor size, perineural infiltration and vascular infiltration).

**Table 1:** Patient characteristics and clinicopathological variables, and their prognostic value for biochemical failure, clinical failure and prostate cancer death in 535 prostate cancer patients (univariate analyses; log rank test).

Characteristic	Patients (n)	Patients(%)	BF (200 events)		CF (56 events)		PCD(18 events)	
			5-year EFS (%)	p	10-year EFS (%)	p	10-year EFS (%)	p
**Age**				0.237		**0.038**		0.404
≤ 65 years	357	67	77		94		98	
> 65 years	178	33	70		91		98	
**pT-stage**				**<0.001**		**<0.001**		**<0.001**
pT2	374	70	83		97		99	
pT3a	114	21	61		87		98	
pT3b	47	9	43		74		91	
**Preop PSA**				**<0.001**		**0.029**		**0.003**
PSA<10	308	57	81		95		99	
PSA>10	221	42	68		89		97	
Missing	6	1	-		-			
**Gleason**				**<0.001**		**<0.001**		**<0.001**
3+3	183	34	83		98		99	
3+4	219	41	77		94		99	
4+3	81	15	70		90		96	
4+4	17	4	58		86		94	
≥9	35	6	37		65		90	
**Tumor Size**				**<0.001**		**0.002**		0.085
0–20 mm	250	47	83		96		99	
>20 mm	285	53	68		90		97	
**PNI**				**<0.001**		**<0.001**		**<0.001**
No	401	75	80		96		99	
Yes	134	25	60		83		95	
**PSM**				**0.049**		0.198		0.843
No	249	47	81		96		98	
Yes	286	53	69		90		98	
**Non-apical PSM**				**<0.001**		**<0.001**		**0.022**
No	381	71	82		96		99	
Yes	154	29	57		85		96	
**Apical PSM**				0.063		0.427		0.128
No	325	61	74		92		98	
Yes	210	39	77		93		99	
**LVI**				**<0.001**		**<0.001**		**<0.001**
No	492	92	77		95		99	
Yes	43	8	47		69		90	
**PRL-3 expression in tumor cytoplasm**				0.131		**0.044**		**0.041**
Low expression	236	44	76		95		99	
High expression	161	30	72		92		96	
Missing	138	26	-		-		-	
**PRL-3 expression in tumor nucleus**				0.123		0.819		0.491
Low expression	225	42	71		94		98	
High expression	172	32	79		94		98	
Missing	138	26	-		-			

Abbreviations: BF = biochemical failure; CF = Clinical failure; LVI = lymphovascular infiltration; PCD = Prostate cancer death; PNI = Perineural infiltration; Preop = preoperative; PSA = Prostate specific antigen; PSM = Positive surgical margin

“Missing” corresponds to missing evaluable tumor tissue for this patient in our TMA cores.

### Multivariate analyses

For the multivariate analyses we entered all clinicopathological variables with a p <0.10 from the univariate analyses in addition to the prognostically significant PRL-3 variable, cytoplasmic tumor cell expression of PRL-3. These variables are in bold in Table 1 and were entered in the three models according to different survival end points; BFFS, CFFS and PCDFS. However, for the last model with PCDFS there were only 18 events, which according to stringent statistical procedures, do not allow more than three variables to be entered. In all models (Table 2) cytoplasmatic PRL-3 expression in tumor cells was an independent prognosticator for poor event-free survival (BFFS, HR = 1.53, CI95% 1.10–2.13, p = 0.012; CFFS, HR = 2.41, CI95% 1.17–4.98, p = 0.017; PCDFS, HR = 3.99, CI95% 1.21–13.1, p = 0.023).

**Table 2:** Multivariate analyses of factors with a p < 0.10 in univariate analyses (see Table 1) for all patients (Cox regression, backward conditional). Significant p-values in bold (threshold p ≤ 0.05).

Characteristic	BF (200 events)			CF (56 events)			PCD (18 events)*		
	HR	CI95%	p	HR	CI95%	p	HR	CI95%	p
**Age**	NE			NS			NE		
**pT-stage**			**<0.001**	NS			NE		
pT2	1								
pT3a	1.56	1.04–2.33	0.031						
pT3b	3.14	1.45–3.97	**<0.001**						
**Tumor Size**	NS			NS			NE		
0–20 mm									
>20 mm									
**Preop PSA**				NS					**0.018**
PSA<10	1						1		
PSA>10	1.49	1.07–2.11	**0.02**				4.74	1.30–17.3	
**ISUP grade (Gleason)**	NS					**0.003**	NS		
1 (3+3)				1					
2 (3+4)				2.74	0.75–10.1	0.127			
3 (4+3)				5.39	1.40–20.7	**0.014**			
4 (4+4)				10.7	2.11–54.4	**0.004**			
5 (≥9)				10.3	2.59–41.3	**0.001**			
**PNI**			**0.003**	NS					**0.002**
No	1						1		
Yes	1.74	1.21–2.49					5.95	1.94–18.3	
**LVI**	NS					**0.007**	NE		
No				1					
Yes				3.35	1.38–8.13				
**Non-apical PSM**			**0.019**	NS			NE		
No	1								
Yes	1.53	1.07–2.19							
**PRL-3 expression in tumor cytoplasm**			**0.012**			**0.017**			**0.023**
Low expression	1			1			1		
High expression	1.53	1.10–2.13		2.41	1.17–4.98		3.99	1.21–13.1	

Abbreviations; BF = biochemical failure; CF = Clinical failure; LVI = lymphovascular infiltration; NE = not entered, due to non-significance in the univariate analyses; NS = not significant, the characteristic is removed by the backward conditional analysis due to insignificance; PCD = Prostate cancer death; PNI = Perineural infiltration; Preop = preoperative; PRL-3 = Phosphatase related to the liver- 3; PSA = Prostate specific antigen; PSM = Positive surgical margin

*Due to the low number of events in the PCD model only three variables could be entered in the model. We therefore did a careful analysis to select only the two variables other than PRL-3 that where truly independent by performing initial multiple enter analyses.

## Discussion

In our large retrospective PC cohort, we found high cytoplasmic tumor cell expression of PRL-3 to be independently associated to all investigated endpoints; BF, CF and pPCdeath.

This is the first study to evaluate the prognostic impact of PRL-3 in PC. It follows a functional study on the role of PRL-3 in PC [22] and thereby further verifies its significance in PC. In addition to a functional study-based hypothesis, strengths of this study are the large well-defined cohort with long follow-up, a validated antibody using a well-adopted method (IHC), and consistent results across several endpoints. Weaknesses are the retrospective design and the lack of a training set to determine cut-offs for validation.

The many previous studies with different methods for PRL-3 detection have implicated its role in cancer, mostly demonstrating associations between high expression and poor prognosis. Associations between high protein expression and poor prognosis have been found in a variety of cancers; breast cancer[10, 26–29], colorectal cancer[7, 9, 30], gastric cancer[13, 31–34], hepatocellular carcinoma[35], cholangiocarcinoma[36], nasopharyngeal carcinoma[37], ovarian cancer[38] and adenoid cystic carcinoma [16], although there have been negative studies too [39].

Studies points to an important role of PRL-3 in cancer progression and metastasis. Initially PRL-3 was proposed as a phosphatase for metastasis[40], but multiple pathways and mechanisms have been implied to exert the effects of high PRL-3 expression. PRL-3 is a member of the PRL protein tyrosine phosphatase family and is the most studied of these so far [6, 41]. All (PRL-1, PRL-2 and PRL-3) promote proliferation, migration, invasion and metastasis[6]. PRL-3 has specifically been implicated in activation of acknowledged cancer progression pathways like phosphatidylinositol-3 kinase[42], regulating mTOR activation[43], Src tyrosine protein kinase[44, 45], epidermal growth factor receptor (EGFR)[46], and ERK[15]. Regulation of PRL-3 is found at several levels (transcriptional, translational and post translational) and is mediated by several factors such as p53, TGFβ, STAT3, VEGF, Snail, PCBP1, Src etc [6, 47]. Hence, its function is complex and probably finely tuned within specific compartments.

In PC, its function has been studied in a few studies. A thorough exploration by members of our group [22] revealed several novel PC-specific findings. PRL-3 was found to be expressed at higher levels in PC tissue than in normal prostate tissue, and was ranked among the genes most differentially expressed between cancerous and benign prostate tissue. In PC cell lines, PRL-3 was present and gene amplication was found to be a possible explanation. Further, inhibition of PRL-3 hampered the PC cell lines’ ability to proliferate, reduced their survival and decreased cell migration. In a small exploration of primary PC tissue and corresponding affected lymph nodes from four patients, they found no difference in expression between the metastases and primary tumor. Taken together, PRL-3 expression is probably an early event in PC tumor progression, and inhibition of PRL-3 causes reduction of pathogenic properties like migration and growth while increasing apoptosis.

This study have implications for future biomarker research in PC. In contrast to many other biomarker studies in PC, PRL-3 was significant for all clinically relevant endpoints, and it should have priority for further validation. In particular since previous biomarker studies in PC only have significant results for BF. In addition, PRL-3 has consistently been found associated with poor prognosis also in several other malignancies. Besides, PRL-3 may have potential as a therapeutic target. The findings from functional studies in various cancers including PC indicates PRL-3 to be an attractive target.

There are currently no ongoing clinical studies targeting PRL-3. However, over the last decade multiple novel PRL-3 inhibitors have been developed[48–53] and several natural occurring compounds are found to have PRL-3-inhibitory properties[54–58], both with clear *in vitro* effects on various types of cancer cells. *In vitro* studies have also investigated effects of PRL-3 inhibition on PC cells. In an explorative study on the effect of curcumin, this agent decreased PRL-3 mRNA levels in PC3 cells[59]. A marine macrolide (halichondramide) had anti-metastatic activity in highly metastatic PC3 human PC cells due to PRL-3 inhibition. The first chimeric antibody targeting PRL-3 was engineered in 2012[60]. Recently, a humanized antibody against PRL-3 (PRL3-zumab) was generated and proved effective towards human gastric cancer cells[20]. Interestingly, effects were associated with PRL-3 positive cells, suggesting expression of PRL-3 to be a possible predictive biomarker for future PRL-3 directed therapy. Our findings of RPL-3 to be primarily expressed in neoplastic and not stromal PC cells support the idea of specific tumor effects by inhibition. Though, this remains to be tested in preclinical studies prior to early phase clinical studies.

## Conclusions

This is the first study to address the prognostic impact of PRL-3 in PC. We have verified our hypothesis that high tumor cell expression of PRL-3 is a strong independent predictor for clinically relevant PC endpoints such as BF, CF and PC death. These results strongly suggest PRL-3 as a prognostic biomarker in PC, although further validation is needed. Based on the results from this study, PRL-3 is suggested as a potential therapeutic target due to expression mostly in cancer cells.

## Supporting information

S1 Fig Immunohistochemical expression of PRL-3 in whole section. PRL-3 staining in a whole section illustrating nuclear and cytoplasmic expression in both malignant and benign epithelium.
(JPG)

S1 File Database file. This is the SPSS database file with scoring and survival data for the patients within this cohort.
(SAV)

## References

[1] Ervik M, Lam F, Ferlay J, Mery L, Soerjomataram I, Bray F. Cancer Today Lyon, France: International Agency for Research on Cancer.; 2016 [updated 2016. http://gco.iarc.fr/today.

[2] van der Kwast TH, Roobol MJ. Defining the threshold for significant versus insignificant prostate cancer. Nat Rev Urol. 2013;10(8):473–82. doi: 10.1038/nrurol.2013.112 23712205 10.1038/nrurol.2013.112

[3] Gronberg H, Adolfsson J, Aly M, Nordstrom T, Wiklund P, Brandberg Y, et al Prostate cancer screening in men aged 50–69 years (STHLM3): a prospective population-based diagnostic study. Lancet Oncol. 2015;16(16):1667–76. doi: 10.1016/S1470-2045(15)00361-7 26563502 10.1016/S1470-2045(15)00361-7

[4] Epstein JI, Egevad L, Amin MB, Delahunt B, Srigley JR, Humphrey PA. The 2014 International Society of Urological Pathology (ISUP) Consensus Conference on Gleason Grading of Prostatic Carcinoma: Definition of Grading Patterns and Proposal for a New Grading System. Am J Surg Pathol. 2016;40(2):244–52. doi: 10.1097/PAS.0000000000000530 26492179 10.1097/PAS.0000000000000530

[5] Saha S, Bardelli A, Buckhaults P, Velculescu VE, Rago C, St C B, et al A phosphatase associated with metastasis of colorectal cancer. Science. 2001;294(5545):1343–6. doi: 10.1126/science.1065817 11598267 10.1126/science.1065817

[6] Rubio T, Kohn M. Regulatory mechanisms of phosphatase of regenerating liver (PRL)-3. Biochemical Society Transactions. 2016;44(5):1305–12. doi: 10.1042/BST20160146 27911713 10.1042/BST20160146 PMC5095905

[7] Peng L, Ning J, Meng L, Shou C. The association of the expression level of protein tyrosine phosphatase PRL-3 protein with liver metastasis and prognosis of patients with colorectal cancer. J Cancer Res Clin Oncol. 2004;130(9):521–6. doi: 10.1007/s00432-004-0563-x 15133662 10.1007/s00432-004-0563-x PMC12161869

[8] Wang Y, Li ZF, He J, Li YL, Zhu GB, Zhang LH, et al Expression of the human phosphatases of regenerating liver (PRLs) in colonic adenocarcinoma and its correlation with lymph node metastasis. Int J Colorectal Dis. 2007;22(10):1179–84. doi: 10.1007/s00384-007-0303-1 17440740 10.1007/s00384-007-0303-1

[9] Mollevi DG, Aytes A, Padulles L, Martinez-Iniesta M, Baixeras N, Salazar R, et al PRL-3 is essentially overexpressed in primary colorectal tumours and associates with tumour aggressiveness. Br J Cancer. 2008;99(10):1718–25. doi: 10.1038/sj.bjc.6604747 19002188 10.1038/sj.bjc.6604747 PMC2584959

[10] Radke I, Gotte M, Kersting C, Mattsson B, Kiesel L, Wulfing P. Expression and prognostic impact of the protein tyrosine phosphatases PRL-1, PRL-2, and PRL-3 in breast cancer. Br J Cancer. 2006;95(3):347–54. doi: 10.1038/sj.bjc.6603261 16832410 10.1038/sj.bjc.6603261 PMC2360632

[11] Ma Y, Li B. Expression of phosphatase of regenerating liver-3 in squamous cell carcinoma of the cervix. Med Oncol. 2011;28(3):775–80. doi: 10.1007/s12032-010-9514-3 20364335 10.1007/s12032-010-9514-3

[12] Miskad UA, Semba S, Kato H, Matsukawa Y, Kodama Y, Mizuuchi E, et al High PRL-3 expression in human gastric cancer is a marker of metastasis and grades of malignancies: an in situ hybridization study. Virchows Arch. 2007;450(3):303–10. doi: 10.1007/s00428-006-0361-8 17235563 10.1007/s00428-006-0361-8

[13] Li ZR, Wang Z, Zhu BH, He YL, Peng JS, Cai SR, et al Association of tyrosine PRL-3 phosphatase protein expression with peritoneal metastasis of gastric carcinoma and prognosis. Surg Today. 2007;37(8):646–51. doi: 10.1007/s00595-006-3437-9 17643206 10.1007/s00595-006-3437-9

[14] Polato F, Codegoni A, Fruscio R, Perego P, Mangioni C, Saha S, et al PRL-3 phosphatase is implicated in ovarian cancer growth. Clin Cancer Res. 2005;11(19 Pt 1):6835–9. doi: 10.1158/1078-0432.CCR-04-2357 16203771 10.1158/1078-0432.CCR-04-2357

[15] Ming J, Liu N, Gu Y, Qiu X, Wang EH. PRL-3 facilitates angiogenesis and metastasis by increasing ERK phosphorylation and up-regulating the levels and activities of Rho-A/C in lung cancer. Pathology. 2009;41(2):118–26. doi: 10.1080/00313020802579268 19152186 10.1080/00313020802579268

[16] Dong Q, Ding X, Chang B, Wang H, Wang A. PRL-3 promotes migration and invasion and is associated with poor prognosis in salivary adenoid cystic carcinoma. J Oral Pathol Med. 2016;45(2):111–8. doi: 10.1111/jop.12331 26041460 10.1111/jop.12331 PMC5032974

[17] Fagerli UM, Holt RU, Holien T, Vaatsveen TK, Zhan F, Egeberg KW, et al Overexpression and involvement in migration by the metastasis-associated phosphatase PRL-3 in human myeloma cells. Blood. 2008;111(2):806–15. doi: 10.1182/blood-2007-07-101139 17934070 10.1182/blood-2007-07-101139 PMC2200854

[18] Bessette DC, Qiu D, Pallen CJ. PRL PTPs: mediators and markers of cancer progression. Cancer Metastasis Rev. 2008;27(2):231–52. doi: 10.1007/s10555-008-9121-3 18224294 10.1007/s10555-008-9121-3

[19] Lee JD, Jung H, Min SH. Identification of proteins suppressing the functions of oncogenic phosphatase of regenerating liver 1 and 3. Exp Ther Med. 2016;12(5):2974–82. doi: 10.3892/etm.2016.3722 27882103 10.3892/etm.2016.3722 PMC5103732

[20] Thura M, Al-Aidaroos AQ, Yong WP, Kono K, Gupta A, Lin YB, et al PRL3-zumab, a first-in-class humanized antibody for cancer therapy. JCI Insight. 2016;1(9):e87607 doi: 10.1172/jci.insight.87607 27699276 10.1172/jci.insight.87607 PMC5033845

[21] Feik E, Schweifer N, Baierl A, Sommergruber W, Haslinger C, Hofer P, et al Integrative analysis of prostate cancer aggressiveness. Prostate. 2013;73(13):1413–26. doi: 10.1002/pros.22688 23813660 10.1002/pros.22688

[22] Vandsemb EN, Bertilsson H, Abdollahi P, Storkersen O, Vatsveen TK, Rye MB, et al Phosphatase of regenerating liver 3 (PRL-3) is overexpressed in human prostate cancer tissue and promotes growth and migration. J Transl Med. 2016;14:71 doi: 10.1186/s12967-016-0830-z 26975394 10.1186/s12967-016-0830-z PMC4791872

[23] Andersen S, Richardsen E, Nordby Y, Ness N, Storkersen O, Al-Shibli K, et al Disease-specific outcomes of radical prostatectomies in Northern Norway; a case for the impact of perineural infiltration and postoperative PSA-doubling time. BMC Urol. 2014;14:49 doi: 10.1186/1471-2490-14-49 24929427 10.1186/1471-2490-14-49 PMC4067377

[24] Wang H, Vardy LA, Tan CP, Loo JM, Guo K, Li J, et al PCBP1 suppresses the translation of metastasis-associated PRL-3 phosphatase. Cancer Cell. 2010;18(1):52–62. doi: 10.1016/j.ccr.2010.04.028 20609352 10.1016/j.ccr.2010.04.028

[25] Li J, Guo K, Koh VW, Tang JP, Gan BQ, Shi H, et al Generation of PRL-3- and PRL-1-specific monoclonal antibodies as potential diagnostic markers for cancer metastases. Clin Cancer Res. 2005;11(6):2195–204. doi: 10.1158/1078-0432.CCR-04-1984 15788667 10.1158/1078-0432.CCR-04-1984

[26] Min L, Ma RL, Yuan H, Liu CY, Dong B, Zhang C, et al Combined expression of metastasis related markers Naa10p, SNCG and PRL-3 and its prognostic value in breast cancer patients. Asian Pac J Cancer Prev. 2015;16(7):2819–26. 25854368 10.7314/apjcp.2015.16.7.2819

[27] den HP, Rawls K, Tsimelzon A, Shepherd J, Mazumdar A, Hill J, et al Phosphatase PTP4A3 Promotes Triple-Negative Breast Cancer Growth and Predicts Poor Patient Survival. Cancer Res. 2016;76(7):1942–53. doi: 10.1158/0008-5472.CAN-14-0673 26921331 10.1158/0008-5472.CAN-14-0673 PMC4873402

[28] Hao RT, Zhang XH, Pan YF, Liu HG, Xiang YQ, Wan L, et al Prognostic and metastatic value of phosphatase of regenerating liver-3 in invasive breast cancer. J Cancer Res Clin Oncol. 2010;136(9):1349–57. doi: 10.1007/s00432-010-0786-y 20140626 10.1007/s00432-010-0786-y PMC11827964

[29] Wang L, Peng L, Dong B, Kong L, Meng L, Yan L, et al Overexpression of phosphatase of regenerating liver-3 in breast cancer: association with a poor clinical outcome. Ann Oncol. 2006;17(10):1517–22. doi: 10.1093/annonc/mdl159 16873432 10.1093/annonc/mdl159

[30] Xing X, Peng L, Qu L, Ren T, Dong B, Su X, et al Prognostic value of PRL-3 overexpression in early stages of colonic cancer. Histopathology. 2009;54(3):309–18. doi: 10.1111/j.1365-2559.2009.03226.x 19236507 10.1111/j.1365-2559.2009.03226.x

[31] Xing X, Lian S, Hu Y, Li Z, Zhang L, Wen X, et al Phosphatase of regenerating liver-3 (PRL-3) is associated with metastasis and poor prognosis in gastric carcinoma. J Transl Med. 2013;11:309 doi: 10.1186/1479-5876-11-309 24330843 10.1186/1479-5876-11-309 PMC3878674

[32] Ooki A, Yamashita K, Kikuchi S, Sakuramoto S, Katada N, Watanabe M. Phosphatase of regenerating liver-3 as a prognostic biomarker in histologically node-negative gastric cancer. Oncol Rep. 2009;21(6):1467–75. 19424625 10.3892/or_00000376

[33] Dai N, Lu AP, Shou CC, Li JY. Expression of phosphatase regenerating liver 3 is an independent prognostic indicator for gastric cancer. World J Gastroenterol. 2009;15(12):1499–505. doi: 10.3748/wjg.15.1499 19322925 10.3748/wjg.15.1499 PMC2665146

[34] Wang Z, Cai SR, He YL, Zhan WH, Chen CQ, Cui J, et al High expression of PRL-3 can promote growth of gastric cancer and exhibits a poor prognostic impact on patients. Ann Surg Oncol. 2009;16(1):208–19. doi: 10.1245/s10434-008-0214-6 19009246 10.1245/s10434-008-0214-6

[35] Mayinuer A, Yasen M, Mogushi K, Obulhasim G, Xieraili M, Aihara A, et al Upregulation of protein tyrosine phosphatase type IVA member 3 (PTP4A3/PRL-3) is associated with tumor differentiation and a poor prognosis in human hepatocellular carcinoma. Ann Surg Oncol. 2013;20(1):305–17. doi: 10.1245/s10434-012-2395-2 23064776 10.1245/s10434-012-2395-2 PMC3528959

[36] Xu Y, Zhu M, Zhang S, Liu H, Li T, Qin C. Expression and prognostic value of PRL-3 in human intrahepatic cholangiocarcinoma. Pathol Oncol Res. 2010;16(2):169–75. doi: 10.1007/s12253-009-9200-y 19757198 10.1007/s12253-009-9200-y

[37] Zhou J, Wang S, Lu J, Li J, Ding Y. Over-expression of phosphatase of regenerating liver-3 correlates with tumor progression and poor prognosis in nasopharyngeal carcinoma. Int J Cancer. 2009;124(8):1879–86. doi: 10.1002/ijc.24096 19101992 10.1002/ijc.24096

[38] Ren T, Jiang B, Xing X, Dong B, Peng L, Meng L, et al Prognostic significance of phosphatase of regenerating liver-3 expression in ovarian cancer. Pathol Oncol Res. 2009;15(4):555–60. doi: 10.1007/s12253-009-9153-1 19247814 10.1007/s12253-009-9153-1

[39] Ustaalioglu BB, Bilici A, Barisik NO, Aliustaoglu M, Vardar FA, Yilmaz BE, et al Clinical importance of phosphatase of regenerating liver-3 expression in breast cancer. Clin Transl Oncol. 2012;14(12):911–22. doi: 10.1007/s12094-012-0880-5 22855168 10.1007/s12094-012-0880-5

[40] Sager JA, Benvenuti S, Bardelli A. PRL-3: a phosphatase for metastasis? Cancer Biol Ther. 2004;3(10):952–3. 15467431 10.4161/cbt.3.10.1115

[41] Rios P, Li X, Kohn M. Molecular mechanisms of the PRL phosphatases. FEBS J. 2013;280(2):505–24. doi: 10.1111/j.1742-4658.2012.08565.x 22413991 10.1111/j.1742-4658.2012.08565.x

[42] Jiang Y, Liu XQ, Rajput A, Geng L, Ongchin M, Zeng Q, et al Phosphatase PRL-3 is a direct regulatory target of TGFbeta in colon cancer metastasis. Cancer Res. 2011;71(1):234–44. doi: 10.1158/0008-5472.CAN-10-1487 21084277 10.1158/0008-5472.CAN-10-1487 PMC3064433

[43] Ye Z, Al-Aidaroos AQ, Park JE, Yuen HF, Zhang SD, Gupta A, et al PRL-3 activates mTORC1 in Cancer Progression. Sci Rep. 2015;5:17046 doi: 10.1038/srep17046 26597054 10.1038/srep17046 PMC4657013

[44] Liang F, Luo Y, Dong Y, Walls CD, Liang J, Jiang HY, et al Translational control of C-terminal Src kinase (Csk) expression by PRL3 phosphatase. J Biol Chem. 2008;283(16):10339–46. doi: 10.1074/jbc.M708285200 18268019 10.1074/jbc.M708285200 PMC2447647

[45] Abdollahi P, Vandsemb EN, Hjort MA, Misund K, Holien T, Sponaas AM, et al Src Family Kinases are Regulated in Multiple Myeloma Cells by Phosphatase of Regenerating Liver-3. Mol Cancer Res. 2016. 10.1158/1541-7786.MCR-16-0212 27698077

[46] Al-Aidaroos AQ, Yuen HF, Guo K, Zhang SD, Chung TH, Chng WJ, et al Metastasis-associated PRL-3 induces EGFR activation and addiction in cancer cells. J Clin Invest. 2013;123(8):3459–71. doi: 10.1172/JCI66824 23867504 10.1172/JCI66824 PMC4011027

[47] Xu J, Cao S, Wang L, Xu R, Chen G, Xu Q. VEGF promotes the transcription of the human PRL-3 gene in HUVEC through transcription factor MEF2C. PLoS One. 2011;6(11):e27165 doi: 10.1371/journal.pone.0027165 22073279 10.1371/journal.pone.0027165 PMC3206935

[48] Ahn JH, Kim SJ, Park WS, Cho SY, Ha JD, Kim SS, et al Synthesis and biological evaluation of rhodanine derivatives as PRL-3 inhibitors. Bioorg Med Chem Lett. 2006;16(11):2996–9. doi: 10.1016/j.bmcl.2006.02.060 16530413 10.1016/j.bmcl.2006.02.060

[49] Min G, Lee SK, Kim HN, Han YM, Lee RH, Jeong DG, et al Rhodanine-based PRL-3 inhibitors blocked the migration and invasion of metastatic cancer cells. Bioorg Med Chem Lett. 2013;23(13):3769–74. doi: 10.1016/j.bmcl.2013.04.092 23726031 10.1016/j.bmcl.2013.04.092

[50] Daouti S, Li WH, Qian H, Huang KS, Holmgren J, Levin W, et al A selective phosphatase of regenerating liver phosphatase inhibitor suppresses tumor cell anchorage-independent growth by a novel mechanism involving p130Cas cleavage. Cancer Res. 2008;68(4):1162–9. doi: 10.1158/0008-5472.CAN-07-2349 18281492 10.1158/0008-5472.CAN-07-2349

[51] Hoeger B, Diether M, Ballester PJ, Kohn M. Biochemical evaluation of virtual screening methods reveals a cell-active inhibitor of the cancer-promoting phosphatases of regenerating liver. Eur J Med Chem. 2014;88:89–100. doi: 10.1016/j.ejmech.2014.08.060 25159123 10.1016/j.ejmech.2014.08.060 PMC4255093

[52] Salamoun JM, McQueeney KE, Patil K, Geib SJ, Sharlow ER, Lazo JS, et al Photooxygenation of an amino-thienopyridone yields a more potent PTP4A3 inhibitor. Org Biomol Chem. 2016;14(27):6398–402. doi: 10.1039/c6ob00946h 27291491 10.1039/c6ob00946h PMC4935606

[53] Gari HH, Gearheart CM, Fosmire S, DeGala GD, Fan Z, Torkko KC, et al Genome-wide functional genetic screen with the anticancer agent AMPI-109 identifies PRL-3 as an oncogenic driver in triple-negative breast cancers. Oncotarget. 2016;7(13):15757–71. doi: 10.18632/oncotarget.7462 26909599 10.18632/oncotarget.7462 PMC4941275

[54] Choi SK, Oh HM, Lee SK, Jeong DG, Ryu SE, Son KH, et al Biflavonoids inhibited phosphatase of regenerating liver-3 (PRL-3). Nat Prod Res. 2006;20(4):341–6. doi: 10.1080/14786410500463312 16644528 10.1080/14786410500463312

[55] Han YM, Lee SK, Jeong DG, Ryu SE, Han DC, Kim DK, et al Emodin inhibits migration and invasion of DLD-1 (PRL-3) cells via inhibition of PRL-3 phosphatase activity. Bioorg Med Chem Lett. 2012;22(1):323–6. doi: 10.1016/j.bmcl.2011.11.008 22137788 10.1016/j.bmcl.2011.11.008

[56] Moon MK, Han YM, Lee YJ, Lee LH, Yang JH, Kwon BM, et al Inhibitory activities of anthraquinones from Rubia akane on phosphatase regenerating liver-3. Arch Pharm Res. 2010;33(11):1747–51. doi: 10.1007/s12272-010-1106-4 21116777 10.1007/s12272-010-1106-4

[57] Shin Y, Kim GD, Jeon JE, Shin J, Lee SK. Antimetastatic effect of halichondramide, a trisoxazole macrolide from the marine sponge Chondrosia corticata, on human prostate cancer cells via modulation of epithelial-to-mesenchymal transition. Mar Drugs. 2013;11(7):2472–85. doi: 10.3390/md11072472 23860239 10.3390/md11072472 PMC3736435

[58] Stadlbauer S, Rios P, Ohmori K, Suzuki K, Kohn M. Procyanidins Negatively Affect the Activity of the Phosphatases of Regenerating Liver. PLoS One. 2015;10(7):e0134336 doi: 10.1371/journal.pone.0134336 26226290 10.1371/journal.pone.0134336 PMC4520450

[59] Wang L, Shen Y, Song R, Sun Y, Xu J, Xu Q. An anticancer effect of curcumin mediated by down-regulating phosphatase of regenerating liver-3 expression on highly metastatic melanoma cells. Mol Pharmacol. 2009;76(6):1238–45. doi: 10.1124/mol.109.059105 19779032 10.1124/mol.109.059105

[60] Guo K, Tang JP, Jie L, Al-Aidaroos AQ, Hong CW, Tan CP, et al Engineering the first chimeric antibody in targeting intracellular PRL-3 oncoprotein for cancer therapy in mice. Oncotarget. 2012;3(2):158–71. doi: 10.18632/oncotarget.442 22374986 10.18632/oncotarget.442 PMC3326646

