# Supplementary figures and images for: Expression of phosphatase of regenerating liver (PRL)-3, is independently associated with biochemical failure, clinical failure and death in prostate cancer
Source: PLoS One. 2017 Nov 30;12(11):e0189000. doi: 10.1371/journal.pone.0189000 (PMC5708709; doi:10.1371/journal.pone.0189000)

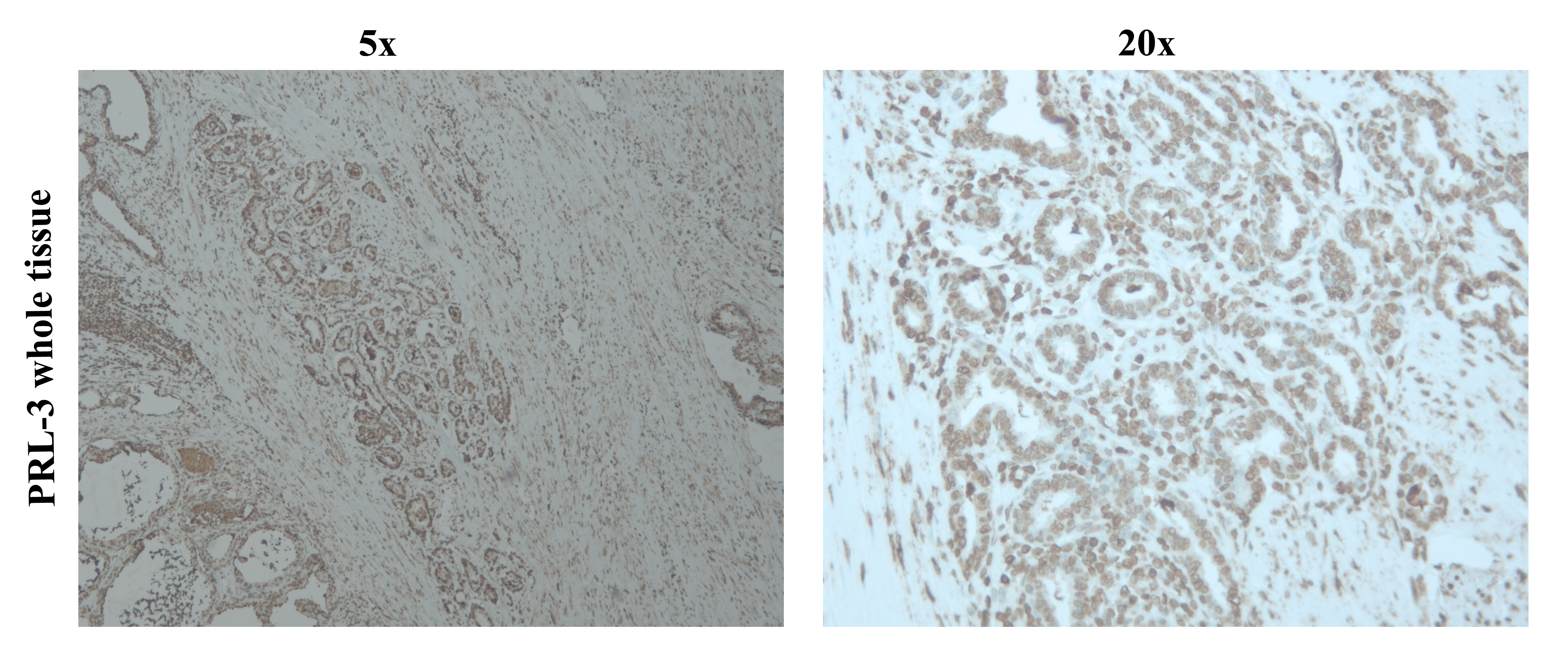

Supplement: S1 Fig — PRL-3 staining in a whole section illustrating nuclear and cytoplasmic expression in both malignant and benign epithelium. (JPG) [file pone.0189000.s001.jpg]
